# Open challenges for microbial network construction and analysis

**DOI:** 10.1038/s41396-021-01027-4

**Published:** 2021-06-09

**Authors:** Karoline Faust

**Affiliations:** KU Leuven, Department of Microbiology, Immunology and Transplantation, Rega Institute for Medical Research, Leuven, Belgium

**Keywords:** Microbiome, Microbial ecology

## Abstract

Microbial network construction is a popular explorative data analysis technique in microbiome research. Although a large number of microbial network construction tools has been developed to date, there are several issues concerning the construction and interpretation of microbial networks that have received less attention. The purpose of this perspective is to draw attention to these underexplored challenges of microbial network construction and analysis.

## Introduction

To date, the microbial composition of thousands of samples from hundreds of ecosystems has been resolved, thanks to high-throughput sequencing [1]. Microbial network construction is a popular exploratory data analysis technique to derive hypotheses from these massive data sets [2]. For instance, it has been applied to identify taxa sharing a common role in an ecosystem such as oxygen-producing cyanobacteria in a seasonally stratified lake [3], to link taxa to a function of interest such as carbon flux in the oceans [4], and to predict biotic interactions [5].

In contrast to networks built for macroorganisms, which are based on observations of biotic interactions between individuals, a microbial network is constructed from a count table obtained from sequencing data. To extract DNA for sequencing, samples need to be homogenized, and thus microhabitats within each sample are aggregated. This means that the microbial network may change with sampling resolution [6]. The steps from the raw reads to the count table come with numerous challenges of their own [7], and unless sequences are derived from RNA, counts do not distinguish between active and dead or dormant cells. Sequences can be grouped into operational taxonomic units in a variety of ways or can be kept separate as amplicon sequence variants [8]. In addition, microbial networks can be constructed at different taxonomic levels. Thus, a node in a microbial network can represent different units depending on the selected sequencing data processing pipeline and taxonomic level, and the choice of the unit also matters for network construction and interpretation. For instance, the challenge of rare taxa discussed below is less pronounced for a microbial network constructed on class level. An edge connecting two nodes representing different units denotes a significant association between the abundances of these units across the samples. Consequently, the biological meaning of an edge in a microbial network is uncertain and requires further analysis and/or experimental validation to be determined.

While new microbial network inference algorithms are published every year, challenges pertaining to data preprocessing, confounding factors, evaluation, and network interpretation are largely ignored. The goal of this perspective is to draw attention to these underexplored challenges of microbial network construction and analysis. For this reason, the list of challenges does not cover technical problems of network construction such as the treatment of compositional data, removal of indirect edges between taxa, and causal inference since they already receive ample attention.

## Challenge #1: Do taxon interactions influence microbial community composition?

The edges in microbial networks are often interpreted as biotic interactions (e.g., cross-feeding of byproducts or competition for nutrients), and several interactions predicted in this manner have been experimentally confirmed [5, 9]. However, interactions may either be absent or too weak to impact community composition, or community composition may have been sampled at spatial or temporal resolutions that are insufficient for ecological interaction detection [6, 10]. In these cases, network inference may still give insights about environmental factors shaping community composition, but if community dynamics are dominated entirely by stochastic processes at the chosen sampling scale, a network is no longer informative, i.e., the correct outcome of network construction should be an empty network. Thus, a quick test to differentiate between stochastic (including neutral) and deterministic community dynamics would prevent time-consuming network construction and misleading interpretation. Even though several tests have been proposed [11–14], they have rarely, if ever, been validated on real communities where the rules governing the dynamics are known. Thus, the first challenge is to develop and evaluate tests for interaction-driven community dynamics and to apply them in the context of microbial network inference.

## Challenge #2: How should abundance data be preprocessed?

Due to differences in extraction, amplification, and sequencing efficiencies, the total read count varies across samples, but is unrelated to cell density and thus carries no biological information. Since taxon abundances covary with total read count, some form of preprocessing is necessary to prevent spurious associations. One such technique is rarefaction, which in essence randomly picks reads from a sample until a predefined lower number of reads has been selected, which is the same for each sample. Since the probability to choose a read from a particular taxon is determined by its proportion in the sample, the original taxon proportions are preserved. Rarefaction has been criticized because it effectively discards a part of the data, lowering the power of microbiome comparisons [15]. However, given the high technical variability of 16S rRNA gene sequencing, this argument does not carry much weight (also, results in ref. [15] have not been reproduced [16]). In fact, repeated rounds of rarefaction have even been used to test the robustness of network inference results [17]. A number of other preprocessing techniques is available besides rarefaction (the simplest of which is the conversion of counts to relative abundances), but there are precious little data on their performance in the context of network construction [17].

Experimentally determined cell densities can also be used to adjust total read counts [18]. Whether this is useful for network construction depends on whether cell densities vary as a result of biotic interactions or due to an external factor that is not of biological interest. For instance, if variations in nutrient concentration change the cell densities but not the species proportions, then varying total cell number (per volume) is a confounder to be removed.

In summary, the second challenge is to compare the performance of different preprocessing techniques across network inference tools to determine which combinations work best.

## Challenge #3: What to do with rare taxa?

**Fig. 1:**
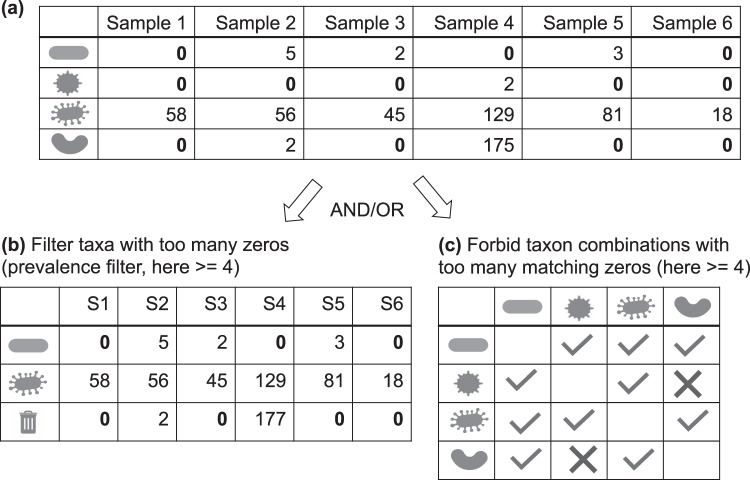
Filtering options for rare taxa. **a** Microbial count tables are usually zero-rich. **b** The prevalence filter removes taxa with too many zeros, while **c** the taxon pair filter forbids associations between taxon pairs with too many matching zeros. The garbage icon represents the sum of the filtered taxa. Both filters necessitate choosing a threshold.

The majority of taxa in sequencing data are only found in very few samples. This means that a large part of sequencing data consists of zeros. In ecological count data, a zero may either represent a true absence or a presence below detection level (i.e., the taxon was there, but its DNA did not make it into the count table). Two taxa with matching zeros across most samples will be strongly associated, but if in truth they vary randomly below detection level, this association will be misleading. There are two filtering approaches to deal with this problem, both of which introduce an arbitrary threshold: the first removes taxa that are present in too few samples (prevalence filter), whereas the second forbids computing an association between taxon pairs when the number of matching zeros is too large (see Fig. 1). Of note, when applying the prevalence filter, the sum of discarded taxa should be kept before further preprocessing steps are carried out, since otherwise the relative abundance of the remaining taxa will be altered.

The rare taxon threshold, whether applied to a single taxon or a taxon pair, has to strike a careful balance: if it is too stringent, it ignores valuable information carried by the zeros, namely that taxon proportions are low in some samples. If on the contrary it is too lenient, it does not resolve the bias caused by too many matching zeros.

Some association measures, such as the Bray Curtis dissimilarity, are designed to ignore matching zeros. However, when too few nonzero value pairs are available, the association is not reliable. Thus, association measures that are robust to matching zeros do not avoid the need to define an arbitrary threshold to deal with zeros. Cougoul et al. recently proposed formulas to compute the number of zeros above which a meaningful test for association is no longer possible, because minimal and maximal association values fall within the confidence interval [19]. This test gives an upper bound for the number of zeros and is a step in the right direction. Depending on the research question, the challenge of rare taxa can also be circumvented by aggregating taxa into higher taxonomic units, e.g., to work on class instead of genus level.

## Challenge #4: How to deal with environmental factors?

**Fig. 2:**
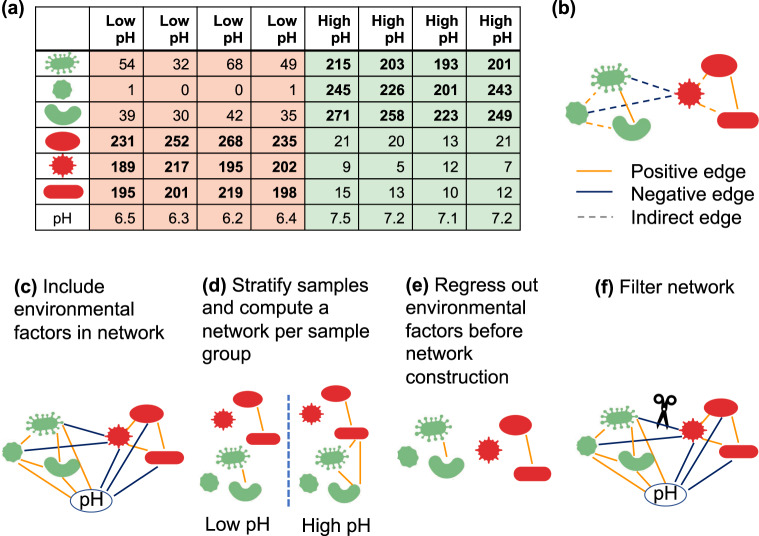
Treatment of environmental heterogeneity. **a** Taxa respond to environmental factors such as pH. **b** A common response to environmental factors introduces indirect edges in the microbial network. To deal with this challenge, **c** environmental factors can be integrated during network construction and considered when interpreting the network, **d** samples can be stratified, either manually or through clustering techniques, and a network constructed per sample group, **e** the impact of environmental factors on taxon abundances can be removed before network construction through regression (often implemented assuming linear environmental response functions), and **f** the network can be filtered to remove indirect edges after construction, for instance, using data processing inequality [24] or network deconvolution [49].

Microbial community composition is strongly influenced by environmental factors such as pH, moisture, oxygen levels, and nutrients. In most systems, these will vary across samples, and microbes will respond to these changes. It is thus difficult to determine whether an edge in a microbial network is due to a common response to an environmental factor (or a third taxon) or represents a direct interaction between two taxa. Several methods exist to deal with the environmental impact (summarized in Fig. 2). The easiest is to include environmental factors as additional nodes and to compute their associations with microbial taxa (Fig. 2c). This is implemented in tools such as CoNet [20] and FlashWeave [21] and in the best cases shows how the environment structures microbial community composition. Another strategy is to split samples into groups, either through sample-wise clustering or according to a key variable such as water depth or health status and to build networks for each sample group separately (e.g., [9, 22], see Fig. 2d). Since the environment is more homogeneous within groups, group-specific networks will have fewer edges due to environmental variation. In extreme cases, taxon presence/absence is entirely due to environmental factors. In these cases, ignoring matching zeros when computing associations, as implemented in FlashWeave’s HE mode, is equivalent to splitting samples into groups. This shows that the problem of environmental heterogeneity is closely linked with the previous challenge of rare taxa; a taxon may only be rare because it belongs to an environment that is underrepresented among the samples. In addition to the environment-as-node and sample-grouping strategies, another method is to regress out environmental factors and to infer associations in the residual abundances that are supposedly free of environmental influence [23] (Fig. 2e). However, many species respond nonlinearly to environmental parameters, i.e., they have an optimal range and decline in growth when the parameter changes beyond that range. Although regression can be extended to handle such nonlinearities, this increases the risk of overfitting the data. Finally, environmentally induced indirect edges can be filtered after network construction (Fig. 2f), for instance, by removing the edge with the lowest mutual information in each fully connected triplet of nodes [9, 24].

Given this range of strategies, which one is the best to deal with environmental influence? The optimal strategy depends on the data and the research question. If the goal is to investigate whether and how the community composition is affected by environmental factors, the environment-as-node strategy can suggest candidate taxa sensitive to specific environmental factors. If in contrast the goal is the inference of biotic interactions, then the environment should be as homogeneous as possible, by stratifying samples or better, by experimental design; the experiment needs to be designed such that biotic interaction detection is possible. If the sampling process aggregates distinct microhabitats, then the network cannot capture biotic interactions specific to a microhabitat.

In case of heterogeneous environments without strong sample-wise clustering, the environment-as-node strategy can be combined with indirect edge filtering or the regression technique can be employed. Since a systematic evaluation of these different techniques is still missing, the fourth challenge is to evaluate different strategies to deal with environmental confounders.

## Challenge #5: What about higher-order interactions (HOIs)?

According to a stringent definition, an HOI is an interaction between a number of species that is altered by an additional species [25]. For instance, if one microbe depends on a molecule secreted by another, and a third microbe produces the same molecule, the cross-feeding relationship between the first two microbes is weakened. HOIs affect community stability and diversity in simulations [26, 27] and were shown to alter host fitness in experiments [28]. HOIs can be detected by measuring growth curves of species pairs and parameterizing a HOI-free model on these data. Deviations of model predictions from community behavior may then indicate the presence of HOIs (e.g., refs. [28, 29]). However, since the HOI-free model may fail to predict observations for other reasons than the presence of HOIs, this approach is not guaranteed to identify HOIs in the narrow sense of modified interactions [25].

Most microbial network construction tools neglect HOIs. Previously, the principle of entropy maximization (finding correlations such that an entropy function is maximized) has been employed to infer HOIs between genes from gene expression data [30]. It is an open question whether entropy maximization could also infer HOIs from microbial abundance data. In presence/absence of data, association rule mining can uncover logical rules that can be interpreted as HOIs. An example for such a rule is a species A that is only found in the presence of two species B and C, for instance, because it needs two cofactors produced by B and C, respectively. In this case, the interaction between A and B or A and C is nonexistent until the arrival of the third species, which can be seen as an extreme case of interaction modification. Although a few association rules involving more than two microbial taxa have been reported previously [31], it is not clear whether these are due to overfitting (a challenge for all HOI inference algorithms), environmental factors, combinations of pair-wise associations, or true HOIs. Finally, visualizing HOIs is not trivial and requires hypergraphs, i.e., networks where an edge connects more than two nodes. Interpreting and analyzing such hypergraphs are additional open challenges of HOIs.

## Challenge #6: How to evaluate microbial network construction in silico?

Evaluations are carried out to assess which tools infer the most accurate networks and to explore how sample number and other data properties affect tool performance. Given the lack of comprehensive biological benchmark data, evaluation of microbial network inference is still to a large extent carried out in silico. Artificial data sets are generated with a number of approaches, ranging from simulations with population models (usually the generalized Lotka–Volterra, e.g., [32]) to statistical approaches that produce multivariate distributions given a desired correlation matrix (NorTA, [33]). In general, tool performance is sensitively dependent on how well the data generation process matches tool assumptions. This is desired if the data generation process is known, but for most microbial communities, it is not precisely known which processes shape taxon abundances. Thus, an evaluation relying on a single data generation method will favor those tools whose assumptions happen to be closest to those behind data generation. To avoid this bias, in silico evaluation needs to employ a range of data simulation procedures incorporating different levels of noise. Another important point is to separate tool development from evaluation since it is difficult for tool developers to evaluate their tools objectively. These criteria are implemented in evaluation frameworks such as the DREAM challenge for gene regulatory network inference [34]. Although a corresponding challenge for microbial network inference is still missing, a few independent evaluations of microbial network inference tools have been carried out to date [17, 35], albeit only the former used several data generation methods. Interestingly, in both evaluations, classical correlation measures such as Pearson and Spearman performed often as well as more sophisticated new network inference algorithms. A possible reason for this surprising outcome may be that new algorithms excel in solving a particular problem for which they were designed, such as inference in highly uneven compositional data, but then perform worse at other problems of equal importance, such as inference in the presence of noise. A DREAM challenge for microbial networks would equip tool developers with more heterogeneous benchmark data and ultimately lead to tools that perform better in more diverse settings.

## Challenge #7: How to benchmark microbial network construction on biological data?

The gold standard for bioinformatics tool benchmarking is an evaluation on biological data for which the result is known. For microbial network inference, this means evaluating inference tools on microbial sequencing data obtained for a community with known interactions. Although such data sets are still rare, a few have been published over the years. For instance, a list of known eukaryotic phytoplankton interactions has been compiled and used for network validation [9]. A large number of microbial interactions have also been validated experimentally for *Arabidopsis* root communities [5].

However, there are several problems when benchmarking network inference on biological data. First, it is not clear whether the list of known interactions is complete and consequently, whether a predicted interaction is wrong or simply was not observed yet. For this reason, interactions observed previously in nature cannot be used to determine the accuracy of network inference tools but only their sensitivity, i.e., the probability that the tool will spot known interactions. This is insufficient to compare tools since a tool can game this criterion by simply reporting as many edges as possible. In contrast, when working with small communities in controlled conditions, all interactions, as well as their absence, can be enumerated (as done in ref. [36]). However, it is then not certain whether these interactions are also relevant in nature. The second problem of benchmarking with biological data is that inferred interactions may differ from expected interactions due to HOIs rather than mistakes in the inference. One way to address this problem is to test for the presence of HOIs as described in the fifth challenge.

In conclusion, communities where interactions are known and sequencing data are available are the gold standard for benchmarking microbial network construction. Since such data sets are still rare, the challenge is an experimental one: to generate many more of these benchmark data to improve tool performance.

## Challenge #8: What can we learn from the hairballs?

Microbial network inference algorithms usually return “hairballs” of densely interconnected taxa that require further analysis to yield testable hypotheses. But despite the wealth of inference tools, only a few analysis tools dedicated to microbial networks have been developed to date [37–39]. Two types of network analysis are particularly informative: data integration and clustering. Networks are particularly suitable for integrating heterogeneous data. Information about microorganisms such as the presence of particular genes, environmental preferences (e.g., pH optima), and known metabolic abilities can be mapped onto nodes, whereas known interactions or results from other inference tools can be mapped onto edges. These additional data help to confirm interactions and to identify indirect edges that result from common responses to a third taxon or environmental factor. In addition, the integration of external data may in some cases suggest an interaction mechanism. For instance, if taxon A has the vitamin B12 pathway and taxon B lacks genes for B12 synthesis, a positive relationship between them may be mediated by vitamin B12 exchange, as described for gut bacteria [40].

Clustering assigns nodes to groups, either de novo or using predefined cluster memberships. In the first case, network cluster algorithms group taxa together that tend to be connected to the same neighbors. Such taxon groups often covary in response to environmental factors such as pH and temperature, and therefore de novo clustering is a means to uncover niche structure. For instance, the members of different clusters in a cheese microbial network respond differently to moisture [37]. In the second case, taxa are assigned to clusters according to prior knowledge, for instance, their plankton function type [9] or their phylum. This simplifies the network, so that the task is no longer to understand relationships between thousands of taxa but between a dozen groups. In both cases, the clusters can also be tested for enrichment of particular taxonomic groups or functions or correlated to metadata, for instance, to carbon flux [4]. Despite their usefulness for network interpretation, there are few software tools carrying out these tasks. Thus, the eighth challenge is to develop more analysis tools tackling data integration and cluster analysis.

## Challenge #9: How to identify core networks?

High-throughput sequencing makes it possible to sequence many instances of an ecosystem or to sequence an ecosystem across different time points. In these cases, the question arises whether the microbial network is preserved across space or time. A straightforward method to answer this question is to construct a microbial network for each sample group representing an area, condition, or a time point separately and then to compute the intersection of these networks. The resulting intersection network only contains edges that are present in all specific networks and can therefore be interpreted as the core network of the ecosystem of interest. There are several issues with the identification of core networks. First, a core network is informative only if it has more edges than expected by chance. However, it is not clear which null model to choose to compute the random expectation, which complicates the interpretation of core networks. Second, edges may only be preserved in a subset of specific networks. If such edges are encountered more frequently than expected by chance, they are still of interest, however they will be missed by a global intersection approach. Thus, the identification of core networks is more challenging than simply computing the global intersection network and deserves dedicated tools.

A disclaimer is needed here: core networks are not identical to the universal networks discussed by Bashan et al. [41]. These authors propose a test for the universality of interaction networks that drive community dynamics in different instances of an ecosystem. Core networks are inferred networks and therefore may contain edges that do not represent interactions. Thus, the existence of a significant core network does not imply that community dynamics is universal.

## Challenge #10 How well do microbial networks represent ecosystems?

Microbial networks are frequently constructed to identify highly connected nodes, so-called hubs. The idea behind hub detection is that these are taxa of special importance for the ecosystem, i.e., keystones in Paine’s sense [42]. This idea makes two important assumptions: first, that hub taxa can be correctly identified by network inference algorithms and second, that they play indeed a special role in the ecosystem. Evaluations testing the first assumption have shown that network inference algorithms do not always identify known hub nodes correctly [2, 32]. In addition, very few hub taxa have been experimentally confirmed to be keystone species (e.g., [43]), so that the validity of the second assumption is currently an open question. This leads to the more general question whether networks represent ecosystems well enough that systems level insights can be gained through network analysis. Assuming that network inference is sufficiently accurate, can network properties such as negative edge percentage, modularity, and network density give useful information about the ecosystem under study? Although several theoretical studies have addressed the impact of network properties on ecosystem stability (e.g., [44–46]), experimental evidence is still scarce and not always in agreement with theoretical expectations (e.g., [47, 48]). Thus, the final challenge is to explore more deeply the link between network and ecosystem properties.

Addressing these challenges will enable microbial ecologists to better distinguish edges representing biotic interactions from others, which in turn will increase the success rate of validation experiments in interaction discovery. In addition, tools for microbial network clustering and data integration will make it easier to identify biologically meaningful taxon groups. Finally, it is to be hoped that a better understanding of the links between network and ecosystem properties will turn network properties from mere numbers into useful information.

In conclusion, if we want to learn more from microbial networks, we need to broaden our research focus beyond inference algorithms and tackle these challenges.

## References

[1] Thompson LR, Sanders JG, McDonald D, Amir A, Ladau J, Locey KJ (2017). A communal catalogue reveals earth’s multiscale microbial diversity. Nature.

[2] Röttjers L, Faust K. From hairballs to hypotheses–biological insights from microbial networks. FEMS Microbiol Rev. 2018;42:761–80. 10.1093/femsre/fuy030 PMC6199531 30085090

[3] Bush T, Diao M, Allen RJ, Sinnige R, Muyzer G, Huisman J (2017). Oxic-anoxic regime shifts mediated by feedbacks between biogeochemical processes and microbial community dynamics. Nat Commun.

[4] Guidi L, Chaffron S, Bittner L, Eveillard D, Larhlimi A, Roux S (2016). Plankton networks driving carbon export in the oligotrophic ocean. Nature.

[5] Durán P, Thiergart T, Garrido-Oter R, Agler M, Kemen E, Schulze-Lefert P (2018). Microbial interkingdom interactions in roots promote *Arabidopsis* survival. Cell.

[6] Armitage DW, Jones SE (2019). How sample heterogeneity can obscure the signal of microbial interactions. ISME J.

[7] McLaren MR, Willis AD, Callahan BJ (2019). Consistent and correctable bias in metagenomic sequencing experiments. eLife.

[8] Bharti R, Grimm DG (2021). Current challenges and best-practice protocols for microbiome analysis. Brief Bioinform.

[9] Lima-Mendez G, Faust K, Henry N, Decelle J, Colin S, Carcillo F, et al. Determinants of community structure in the global plankton interactome. Science. 2015;348:1262073-1–1262073-9. 10.1126/science.1262073 25999517

[10] Fuhrman JA, Cram JA, Needham DM (2015). Marine microbial community dynamics and their ecological interpretation. Nat Rev Microbiol.

[11] Faust K, Bauchinger F, Laroche B, Sd Buyl, Lahti L, Washburne AD (2018). Signatures of ecological processes in microbial community time series. Microbiome.

[12] Stegen JC, Lin X, Fredrickson JK, Chen X, Kennedy DW, Murray CJ (2013). Quantifying community assembly processes and identifying features that impose them. ISME J.

[13] Washburne AD, Burby JW, Lacker D (2016). Novel covariance-based neutrality test of time-series data reveals asymmetries in ecological and economic systems. PLoS Comput Biol.

[14] Zhou J, Ning D (2017). Stochastic community assembly: does it matter in microbial ecology?. Microbiol Mol Biol Rev.

[15] McMurdie PJ, Holmes S (2014). Waste not, want not: Why rarefying microbiome data is inadmissible. PLoS Comput Biol.

[16] Weiss S, Xu ZZ, Peddada S, Amir A, Bittinger K, Gonzalez A (2017). Normalization and microbial differential abundance strategies depend upon data characteristics. Microbiome.

[17] Weiss S, Treuren WV, Lozupone C, Faust K, Friedman J, Deng Y (2016). Correlation detection strategies in microbial data sets vary widely in sensitivity and precision. ISME J.

[18] Vandeputte D, Kathagen G, D’hoe K, Vieira-Silva S, Valles-Colomer M, Sabino J (2017). Quantitative microbiome profiling links gut community variation to microbial load. Nature.

[19] Cougoul A, Bailly X, Vourc’h G, Gasqui P. Rarity of microbial species: in search of reliable associations. PLoS ONE. 2019;14:e0200458. 10.1371/journal.pone.0200458 PMC6420159 30875367

[20] Faust K, Raes J (2016). Conet app: inference of biological association networks using cytoscape. F1000Research.

[21] Tackmann J, Frederico J, Rodrigues M, Mering CV. Rapid inference of direct interactions in large-scale ecological networks from heterogeneous microbial sequencing data. Cell Syst. 2019;9:286–96. 10.1016/j.cels.2019.08.002 31542415

[22] Nagpal S, Singh R, Yadav D, Mande SS (2020). Metagenonets: comprehensive inference and meta-insights for microbial correlation networks. Nucleic Acids Res.

[23] Warton DI, Blanchet FG, O’Hara RB, Ovaskainen O, Taskinen S, Walker SC (2015). So many variables: joint modeling in community ecology. Trends Ecol Evol.

[24] Margolin AA, Nemenman I, Basso K, Wiggins C, Stolovitzky G, Favera RD (2006). Aracne: an algorithm for the reconstruction of gene regulatory networks in a mammalian cellular context. BMC Bioinform.

[25] Billick I, Case TJ (1994). Higher order interactions in ecological communities: what are they and how can they be detected?. Ecology.

[26] Bairey E, Kelsic ED, Kishony R (2016). High-order species interactions shape ecosystem diversity. Nat Commun.

[27] Grilli J, Barabás G, Michalska-Smith MJ, Allesina S (2017). Higher-order interactions stabilize dynamics in competitive network models. Nature.

[28] Gould AL, Zhang V, Lamberti L, Jones EW, Obadia B, Korasidis N (2018). Microbiome interactions shape host fitness. PNAS.

[29] Mickalide H, Kuehn S (2019). Higher-order interaction between species inhibits bacterial invasion of a phototroph-predator microbial community. Cell Syst.

[30] Lezon TR, Banavar JR, Cieplak M, Maritan A, Fedoroff NV (2006). Using the principle of entropy maximization to infer genetic interaction networks from gene expression patterns. PNAS.

[31] Tandon D, Haque MM, Mande SS (2015). Inferring intra-community microbial interaction patterns from metagenomic datasets using associative rule mining techniques. PLoS ONE.

[32] Berry D, Widder S (2014). Deciphering microbial interactions and detecting keystone species with co-occurrence networks. Front Microbiol.

[33] Kurtz ZD, Müller CL, Miraldi ER, Littman DR, Blaser MJ, Bonneau RA (2015). Sparse and compositionally robust inference of microbial ecological networks. PLoS Comput Biol.

[34] Marbach D, Prill RJ, Schaffter T, Mattiussi C, Floreano D, Stolovitzky G (2010). Revealing strengths and weaknesses of methods for gene network inference. PNAS.

[35] Hirano H, Takemoto K (2019). Difficulty in inferring microbial community structure based on co-occurrence network approaches. BMC Bioinform.

[36] Biswas S, McDonald M, Lundberg DS, Dangl JL, Jojic V. Learning microbial interaction networks from metagenomic count data. In: International Conference on Research in Computational Molecular Biology. Springer: Warsaw, Poland; 2015. 10.1089/cmb.2016.0061 27267776

[37] Röttjers L, Faust K. Manta—a clustering algorithm for weighted ecological networks. mSystems. 2020;5:e00903–19. 10.1128/mSystems.00903-19 PMC7029223 32071163

[38] Nagpal S, Baksi KD, Kuntal BK, Mande SS (2020). Netconfer: a web application for comparative analysis of multiple biological networks. BMC Biol.

[39] Kuntal BK, Chandrakar P, Sadhu S, Mande SS (2019). ‘Netshift’: a methodology for understanding ‘driver microbes’ from healthy and disease microbiome datasets. ISME J.

[40] Sharma V, Rodionov DA, Leyn SA, Tran D, Iablokov SN, Ding H (2019). B-vitamin sharing promotes stability of gut microbial communities. Front Microbiol.

[41] Bashan A, Gibson TE, Friedman J, Carey VJ, Weiss ST, Hohmann EL (2016). Universality of human microbial dynamics. Nature.

[42] Paine RT (1966). Food web complexity and species diversity. Am Nat.

[43] Carlström CI, Field CM, Bortfeld-Miller M, Müller B, Sunagawa S, Vorholt JA (2019). Synthetic microbiota reveal priority effects and keystone strains in the *Arabidopsis* phyllosphere. Nat Ecol Evol.

[44] Coyte KZ, Schluter J, Foster KR (2015). The ecology of the microbiome: networks, competition, and stability. Science.

[45] May RM (1972). Will a large complex system be stable?. Nature.

[46] Grilli J, Rogers T, Allesina S (2016). Modularity and stability in ecological communities. Nat Commun.

[47] Jacquet C, Moritz C, Morissette L, Legagneux P, Massol F, Archambault P (2016). No complexity–stability relationship in empirical ecosystems. Nat Commun.

[48] Dalsgaard B, Trøjelsgaard K, González AMM, Nogués-Bravo D, Ollerton J, Petanidou T (2013). Historical climate-change influences modularity and nestedness of pollination networks. Ecography.

[49] Feizi S, Marbach D, Médard M, Kellis M (2013). Network deconvolution as a general method to distinguish direct dependencies in networks. Nat Biotechnol.

